# Tumour stemness and poor clinical outcomes in haemochromatosis patients with hepatocellular carcinoma

**DOI:** 10.1136/jcp-2022-208679

**Published:** 2023-05-30

**Authors:** Daniel M Di Capua, William Shanahan, Michele Bourke, Navneet Ramlaul, Josh Appel, Aoife Canney, Neil G Docherty, Erinn McGrath, Eabha Ring, Fiona Jones, Marie Boyle, Janet McCormack, Tom Gallagher, Emir Hoti, Niamh Nolan, John D Ryan, Diarmaid D Houlihan, Aurelie Fabre

**Affiliations:** 1 HIstopathology, St Vincent's University Hospital, Dublin, Ireland; 2 Liver Unit, St Vincent's University Hospital, Dublin, Ireland; 3 Histopathology, University Hospital Galway, Galway, Ireland; 4 University College Dublin School of Medicine, Dublin, Ireland; 5 Diabetes Complications Research Centre, Conway Institute, University College Dublin, Dublin, Ireland; 6 Reseach Pathology Core, Conway Institute, University College Dublin, Dublin, Ireland; 7 Hepatobiliary and Transplant Surgery, St Vincent's University Hospital, Dublin, Ireland; 8 Hepatology Unit, Beaumont Hospital, Dublin, ireland; 9 RCSI University of Medicine and Health Sciences, Dublin, Ireland

**Keywords:** cancer stem cells, liver, iron

## Abstract

**Aims:**

Patients with haemochromatosis (HFE) are known to have an increased risk of developing hepatocellular carcinoma (HCC). Available data are conflicting on whether such patients have poorer prognosis, and there is lack of data regarding the biology of HFE-HCC. We compared the course of HFE-HCC with a matched non-HFE-HCC control group and examined tumour characteristics using immunohistochemistry.

**Methods:**

In this tertiary care-based retrospective analysis, 12 patients with HFE and 34 patients with alcohol/non-alcoholic steatohepatitis who underwent initially successful curative HCC therapy with ablation or resection were identified from our registry. Time to tumour progression was compared. Resected liver tissue from a separate cohort of 11 matched patients with HFE-HCC and without HFE-HCC was assessed for the expression of progenitor and epithelial–mesenchymal transition markers using immunohistochemistry.

**Results:**

The median follow-up was 24.39 and 24.28 months for patients with HFE-HCC and those without HFE-HCC, respectively (p>0.05). The mean time to progression was shorter in the HFE group compared with the non-HFE group (12.87 months vs 17.78 months; HR 3.322, p<0.05). Patients with HFE-HCC also progressed to more advanced disease by the end of follow-up (p<0.05). Immunohistochemical analysis of matched HFE-HCC and non-HFE-HCC explants demonstrated increased expression of the cancer stem cell markers EpCAM (epithelial cell adhesion molecule) and EpCAM/SALL4 (spalt-like transcription factor 4) coexpression in HFE-HCC specimens (p<0.05). There was a high frequency of combined tumour subtypes within the HFE cohort.

**Conclusions:**

This study demonstrates that the clinical course of patients with HFE-HCC is more aggressive and provides the first data indicating that their tumours have increased expression of progenitor markers. These findings suggest patients with HFE-HCC may need to be considered for transplant at an earlier stage.

**WHAT IS ALREADY KNOWN ON THIS TOPIC**

- Patients with haemochromatosis (HFE) are at increased risk of developing hepatocellular carcinoma (HCC).
- There are conflicting data regarding the clinical course and outcomes of these patients.

**WHAT THIS STUDY ADDS**

- Patients with HFE-HCC have a more aggressive clinical course compared with those without HFE-HCC.
- A biological analysis of HFE-HCC tumours demonstrates increased expression of progenitor markers.

**HOW THIS STUDY MIGHT AFFECT RESEARCH, PRACTICE OR POLICY**

- Patients with potentially curative HCC in the context of HFE should be considered for transplant at an earlier stage, rather than resection or thermal ablation.

## Introduction

 Haemochromatosis (HFE) relates to a syndrome of potentially toxic systemic iron accumulation, which may be genetic or acquired in nature. The C282Y HFE genetic variant leads to a defective HFE protein product, which manifests as impaired sensing of systemic iron levels and reduced secretion of the iron regulatory hormone hepcidin.^1^ Unchecked dietary iron absorption and iron release from recycled red blood cells by macrophages leads to systemic iron accumulation, most prominently in the liver, where fibrosis and cirrhosis may result.

Patients with HFE are at a significantly elevated risk of hepatocellular carcinoma (HCC), particularly men. A recent study from the UK Biobank demonstrated an HR of 10.5 for primary hepatic malignancy in men who are homozygous for the C282Y variant.^2^ Furthermore, reduced survival rates in patients with HFE undergoing orthotopic liver transplant (OLT) for HCC have been reported, particularly with high rates of HCC recurrence.^3 4^ This has led some transplant units to list patients with HFE and HCC for OLT at an earlier stage compared with other patient groups. Robust data to explain the potential mechanisms behind poorer outcomes in HFE-related HCC (HFE-HCC) are lacking.

In recent years, emerging evidence has shown that a subset of HCCs harbouring distinct populations of cancer stem cells (CSC) exhibit a more aggressive phenotype with poorer clinical outcomes compared with conventional HCC.^5^ Epithelial cell adhesion molecule (EpCAM), CD44 and more recently spalt-like transcription factor 4 (SALL4) are specific CSC-related markers that impact the biology of HCC.^6^,^8^ Their expression has been linked to higher tumour grade, lymphovascular invasion, tumour recurrence and poorer survival. Epithelial–mesenchymal transition (EMT) markers epithelial cadherin (e-cadherin), CK18, vimentin and β-catenin are similarly poor prognostic indicators.^9^,^15^ The combination of EMT and CSC properties contributes to a heterogeneous tumour cell population with more potent malignant potential.^9^

In this study we sought to examine the outcomes of patients who develop HCC on a background of HFE. Given that HFE is prevalent in Ireland,^16^ we were able to investigate differences between HFE-HCC and non-HFE-HCC in a select subgroup of patients. Using resected and transplanted specimens, we also sought to examine the biological diversity in HFE-HCC by examining the expression of EMT and CSC-related markers.

## Patients, materials and methods

### Diagnosis of HFE

A diagnosis of HFE was accepted as the cause of the underlying liver damage if any of the following conditions were met:

1. Liver biopsy confirms significant hepatic iron deposition: a hepatic iron index score of ≥1.9 was considered diagnostic of HFE.
2. HFE genotype analysis: a genetic analysis demonstrating C282Y homozygosity. If the patient was a C282Y/H63D compound heterozygote, the presence of raised iron indices (serum ferritin and transferrin saturations >45%), in the absence of features of metabolic syndrome and/or excess alcohol consumption, was considered diagnostic of HFE.
3. Clinical diagnosis: in the absence of criteria 1 or 2, patients referred to our unit from another hepatologist with clinical diagnosis of HFE and receiving phlebotomy.

### Study populations

In the first part of the study, we recruited patients from a prospectively maintained HCC database at St Vincent’s University Hospital (SVUH), Dublin. Patient consent for research was obtained prospectively in the clinic when patients first attended. Patients with HFE and those without HFE (alcohol-related liver disease (ALD) or non-alcoholic steatohepatitis (NASH)) who underwent an initially successful curative treatment (thermal ablation or resection) as their first therapy for HCC were identified and their data analysed in a retrospective fashion. Successful curative therapy was defined as no evidence of HCC in the treatment field on the first follow-up imaging at 3 months following therapy as per protocol. Patient outcomes including time to progression, Barcelona Clinic Liver Cancer (BCLC) stage at the end of the follow-up period and survival were evaluated and compared for both groups.

In the second part of the study, we sought to examine biological differences between patients with HFE-HCC and those without HFE-HCC. All surgical resection specimens over a 10-year period between 2002 and 2012 at the National Liver Transplant Unit at SVUH were identified and reviewed. The histological reports and tissue slides from each of the surgically resected cases were retrieved and assessed. Each HFE-HCC case was matched to a non-HFE-HCC control selected for sex, age, tumour characteristics (tumour number, largest tumour size and tumour aggregate diameter) and preoperative α-fetoprotein (AFP). Eleven matched pairs were included in the histological analysis. The negative control group comprised donor liver tissue obtained at OLT.

### HCC diagnosis (clinical study)

In the context of cirrhosis, non-invasive Liver Imaging Reporting and Data System criteria were used to diagnose HCC as per protocol in our centre.^17^ If biopsy was required to establish the diagnosis, the relevant WHO classification was used.^18 19^

### Clinical study endpoints

The primary endpoint in the study was time to tumour progression. The secondary endpoint was survival. All patients treated in our centre have protocol imaging which involves 3-monthly contrast-enhanced, four-phase CT or MRI for the first year, and 6-monthly contrast-enhanced, four-phase CT or MRI thereafter. Time to progression was measured from the date of therapy to the date of the scan showing imaging evidence of new disease. Treatment response was estimated using the Modified Response Evaluation Criteria in Solid Tumors criteria.^20^

### Immunohistochemistry

A single representative slide was selected from each of the HCC (HFE and non-HFE) and reference liver tissue specimens (n=3). The corresponding archival formalin-fixed, paraffin-embedded tissue block was retrieved and reviewed. HCC was diagnosed using the 2019 WHO classification.^19^

A panel of seven monoclonal mouse IgG_1_ antibodies were selected for detecting EMT and CSC markers (online supplemental table 1). EMT antibodies included e-cadherin (Dako IR059), CK18 (Dako IR618), vimentin (Dako IR630) and β-catenin (Dako IR702). CSC antibodies included EpCAM (Dako IR637), SALL4 (Abcam ab57577) and CD44 (Dako M7082). All antibodies except CD44 and SALL4 were provided in a preoptimised dilution formulation with an optimal incubation time of 20 min. Optimisation of dilution and incubation time for CD44 and SALL4 was conducted using manufacture-specified control tissue (liver and testes, respectively). Optimal SALL4 and CD44 dilution factors were 1:250 and 1:50, respectively, with an incubation time of 30 min (specific automated immunohistochemical protocol details are provided in online supplemental data).

**Table 1:** Patient demographics in the clinical study

	Non-HFE (n=34)	HFE (n=12)	P value
Mean age, years±SD	70.06±8.48	70.95±6.54	0.483
Sex, % (n)			1.000
Male	82.35 (28)	83.33 (10)	
Female	17.65 (6)	16.66 (2)	
Ethnicity, % (n)			1.000
Irish	97.06 (33)	100 (12)	
Other	2.94 (1)	0 (0)	
BCLC stage, % (n)			1.000
0	26.47 (9)	25 (3)	
A	61.76 (21)	66.67 (8)	
B	11.76 (4)	8.33 (1)	
ECOG score, % (n)			1.000
0	97.06 (33)	100 (12)	
1	2.94 (1)	0 (0)	
Cirrhosis, % (n)			0.488
Yes	70.59 (24)	58.33 (7)	
No	29.41 (10)	41.67 (5)	
Child-Pugh (if cirrhotic), % (n)			0.492
A	79.17 (19)	100 (7)	
B	20.83 (5)	0 (0)	
MELD (if cirrhotic)			0.169
Median	8	7	
Range	7–14	6–9	
AFP (kU/L)			0.313
Median	5	4	
Range	1–132	1–310	
Ferritin (µg/L)			0.236
Median	93	73	
Range	10–417	14–2245	
Therapy, % (n)			0.502
Resection	50 (17)	66.67 (8)	
Radiofrequency ablation	50 (17)	33.33 (4)	
Follow-up interval post diagnosis (months)			0.379
Median	24.28	24.39	
Range	7.02–96.00	11.84–60.23	
HFE diagnosis			
Genetic	N/A	6	
Clinical	N/A	5	
Pathological	N/A	1	

AFPα-fetoproteinBCLCBarcelona Clinic Liver CancerECOGEastern Cooperative Oncology GroupHFEhaemochromatosisMELDModel for End-Stage Liver DiseaseN/Anot available

Immunostaining was performed using Dako Autostainer Link 48 according to the manufacturer’s instructions. Employing EnVision FLEX target retrieval solutions (Dako), 4 µm tissue sections were deparaffinised and subjected to antigen retrieval. Following an endogenous enzyme block, sections were subsequently incubated with primary antibody. A secondary reagent, EnVision FLEX+ Mouse LINKER (Dako), was then added, followed by amplification with horseradish peroxidase (HRP)-coated polymer detection reagent, EnVision FLEX/HRP detection reagent (Dako). 3,3′-diaminobenzidine hydrochloride substrate-chromogen visualisation system was employed to highlight the immunohistochemical signal. All sections were counterstained with haematoxylin. To validate specificity, no-antibody positive controls and IgG isotype were included for each antibody.

Digital whole slide scans were obtained using a combined microscope/camera Hamamatsu NanoZoomer-RS C10730-02 with 20× NA 0.75 lens system. Hamamatsu NDP.scan software was used to acquire and export the images.

### Statistical analysis

The baseline demographics and histopathological results of patients in the HFE and non-HFE cohorts were compared using Student’s t-test or Fisher’s exact test as appropriate. Time to progression and overall survival were estimated using the Kaplan-Meier method, and significance was determined via a log-rank test. Patients who did not experience an event were censored at the latest follow-up. A Cox proportional hazards model was used to determine the HR with 95% CI for progression and survival. To determine disease progression at the end of follow-up, the BCLC stage was calculated at the time of the most recent imaging for each patient, and proportions with advanced disease (BCLC B, C or D) in each group were compared using Fisher’s exact test. All tests were two-sided, and p values of <0.05 were considered significant. Statistical analysis was performed using IBM SPSS Statistics 26.

## Results

### Clinical study

Between the years 2012 and 2019 in the study centre, a total of 12 patients with HFE-HCC and 34 without HFE-HCC underwent initially successful curative treatments with either thermal ablation or resection as their first treatment. Baseline demographic and disease characteristics (including sex, age, BCLC stage, Eastern Cooperative Oncology Group score, presence of cirrhosis, severity of liver disease if cirrhotic, ferritin, AFP level at diagnosis, therapy received) were similar between the two groups (table 1).

**Figure 1:**
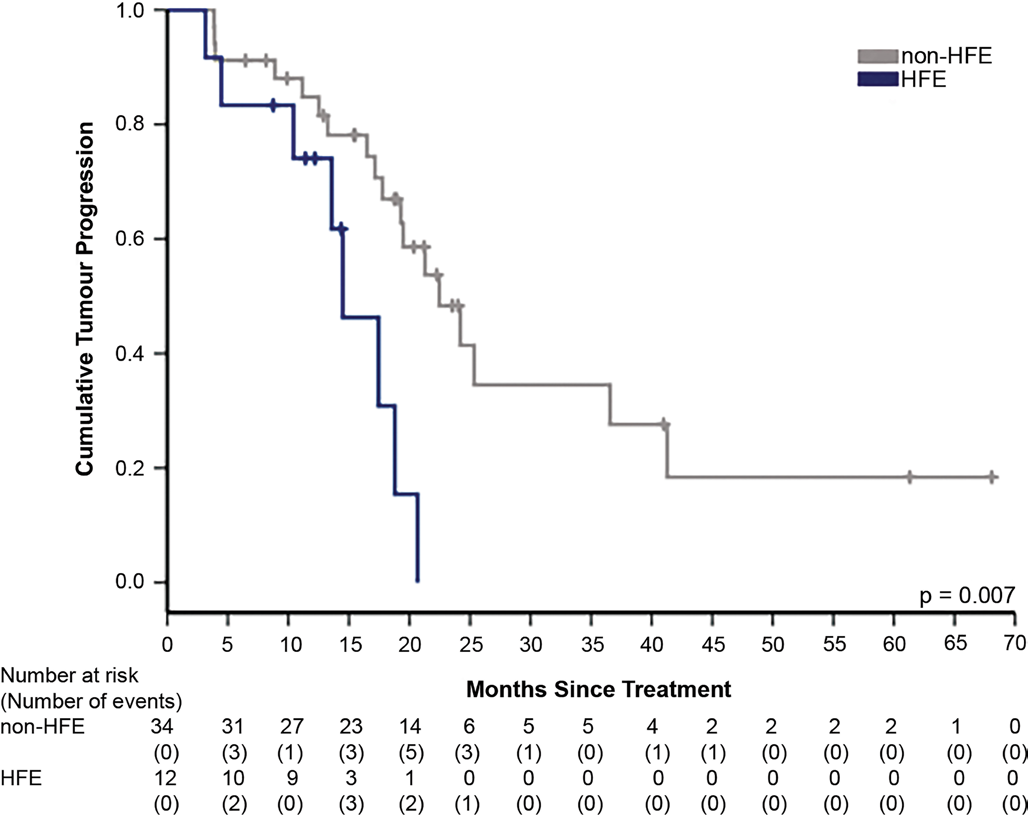
Kaplan-Meier analysis for time to progression in the HFE and non-HFE groups. HCC progression is more rapid in the HFE cohort (p=0.007). HCC, hepatocellular carcinoma; HFE, haemochromatosis.

**Figure 2:**
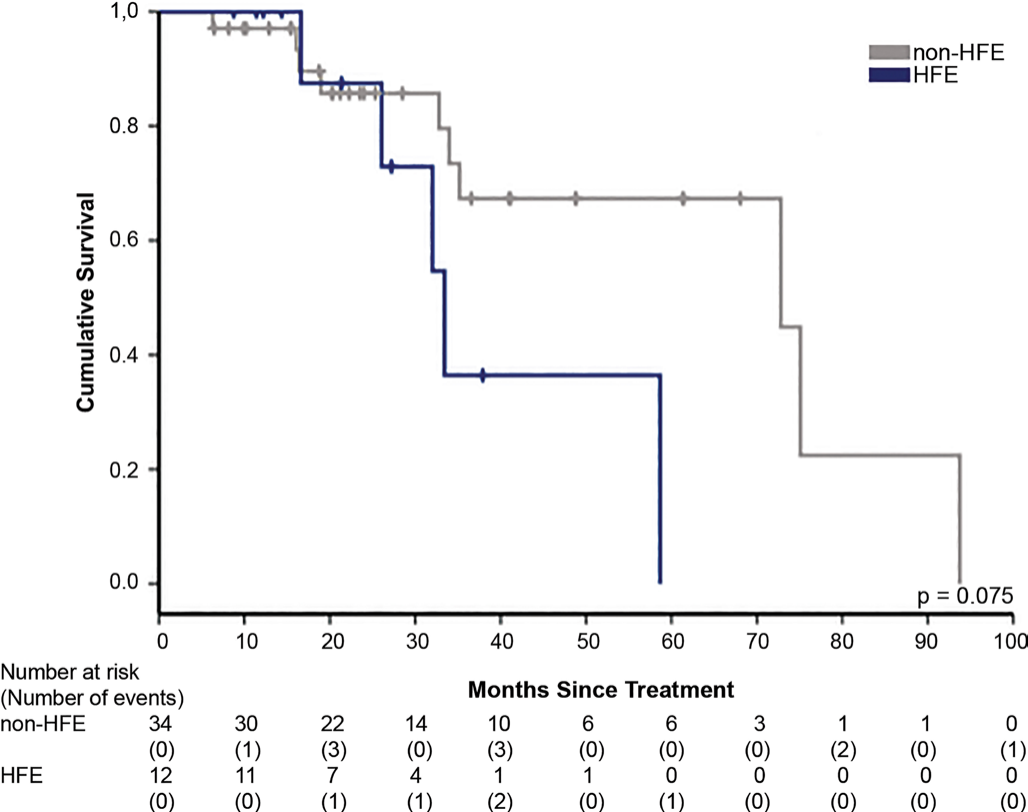
Kaplan-Meier analysis for survival of patients with HFE and those without HFE. There is no statistical difference in cohort survival (p=0.075). HFE, haemochromatosis.

The median patient follow-up was 24.39 months (range: 11.84–60.23) and 24.28 months (range: 7.02–96.00) for patients with HFE and those without HFE, respectively (p=0.379). The Kaplan-Meier analysis demonstrating time to progression for each group is shown in figure 1 (p=0.007). The mean time to progression was significantly shorter in the HFE group compared with the non-HFE group (12.87 months vs 17.78 months; HR for progression in the HFE group 3.322, 95% CI 1.316 to 8.385, p=0.011). Of those with tumour progression, the HFE cohort had a more advanced stage of disease by the end of the follow-up period (BCLC stage ≥B; HFE group 87.5% vs non-HFE group 38.89%, p=0.036). The Kaplan-Meier survival analysis for each group is demonstrated in figure 2 (p=0.075). We did not identify a significant overall survival difference between the groups (cumulative survival rate at 3 years, 36.5% in the HFE group vs 67.3% in the non-HFE group; HR for death, 2.749, 95% CI 0.861 to 8.774, p=0.088).

### Pathology

The two groups had similar baseline demographic and disease characteristics, including age, sex, BCLC stage, presence of lymphovascular invasion, presence of cirrhosis, tumour number and size, AFP level, and ferritin level (table 2). Of the 11 HFE samples, 6 derived from explant specimens (OLT recipients) and 5 from segmentectomies. Two cases underwent transcatheter arterial chemoembolisation (TACE) prior to surgical resection. All the non-HFE specimens were obtained from explant material and five cases had prior TACE. HCC aetiologies in the non-HFE group consisted of NASH (n=5), ALD (n=4), autoimmune hepatitis (n=1) and hepatitis B (n=1). Combined tumour subtypes were observed in 36% of HFE cases (n=4), predominantly combined hepatocellular-cholangiocarcinoma (HCC-CCA) subtype and a single case of HCC with yolk sac differentiation. No such combined tumour subtypes were observed in the non-HFE group (p=0.090).

**Table 2:** Patient demographics in the pathology study

	Non-HFE (n=11)	HFE (n=11)	P value
Mean age, years±SD	61.55±4.01	61.36±7.97	0.947
Sex			1.000
Male	9	9	
Female	2	2	
BCLC stage			0.311
0	1	0	
A	7	10	
B	3	1	
Locoregional therapy			0.361
Yes	5	2	
No	6	9	
Lymphovascular invasion			0.387
Yes	3	6	
No	8	5	
Tumour differentiation			0.805
Well	4	2	
Moderate	6	8	
Poor	1	1	
Cirrhosis			0.635
Yes	9	7	
No	2	4	
Tumour number			1.000
Single	6	6	
Multiple	5	5	
Largest tumour size (cm)			0.431
Median	3.7	3.5	
Range	0.7–7	2–14	
Tumour aggregate diameter (cm)			0.216
Median	4.5	5.8	
Range	0.7–8.9	2–15.5	
AFP (kU/L)			0.381
Median	3.1	12.1	
Range	1–169	1.5–720	
Ferritin (µg/L)			0.062
Median	149.0	585.5	
Range	17–1075	58.5–2084	
HFE diagnosis			
Genetic	N/A	7	
Clinical	N/A	4	

AFPα-fetoproteinBCLCBarcelona Clinic Liver CancerHFEhaemochromatosisN/Anot available

### Immunohistochemical results

**Figure 3:**
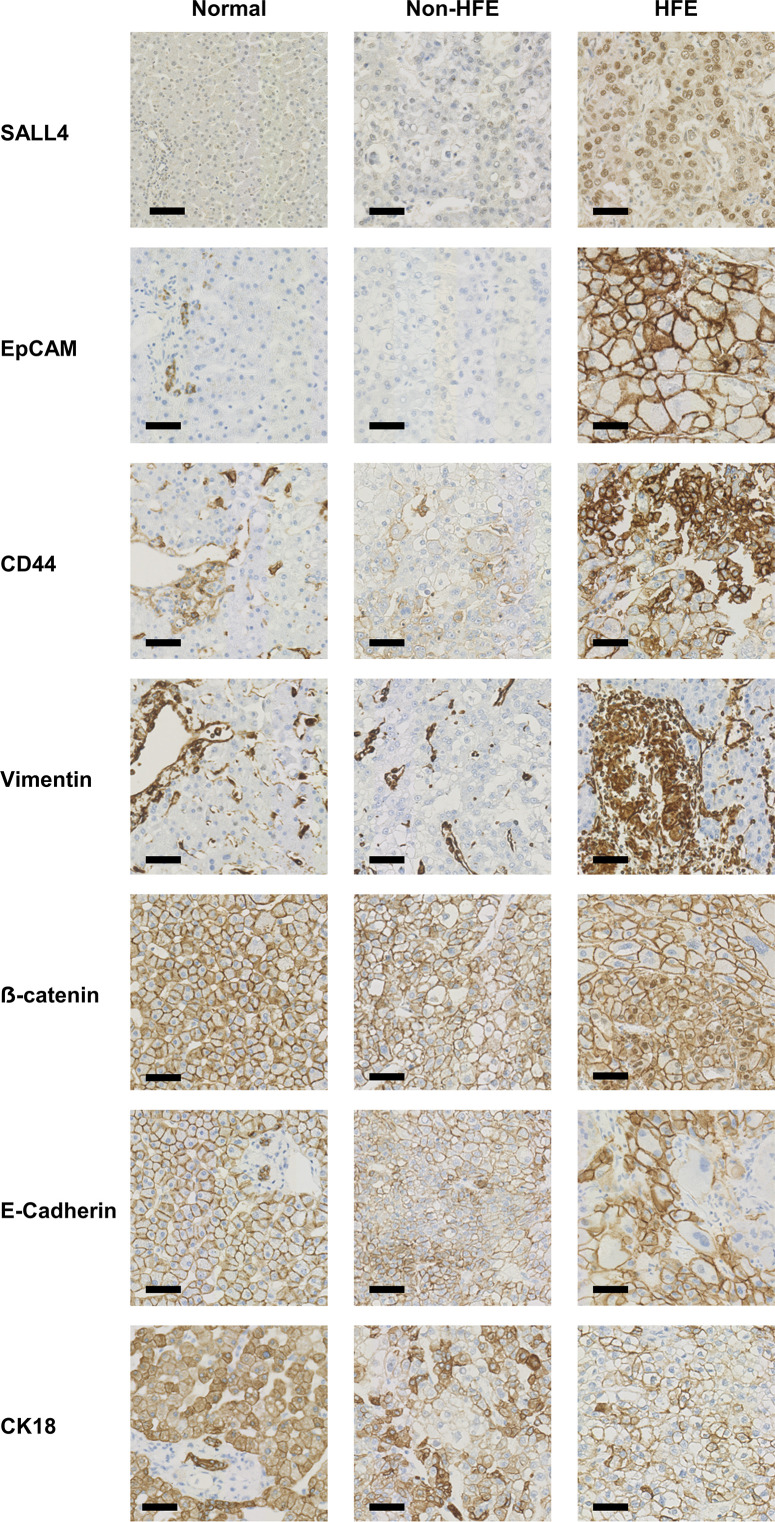
Immunohistochemical staining examples of normal liver tissue, non-HFE-HCC and HFE-HCC with CSC and EMT markers. Scale bar indicates 50 μm. CSC, cancer stem cells; EMT, epithelial–mesenchymal transition; EpCAM, epithelial cell adhesion molecule; HCC, hepatocellular carcinoma; HFE, haemochromatosis; SALL4, spalt-like transcription factor 4.

Immunohistochemical staining results in the HFE and non-HFE groups are shown in table 3. Representative images of the staining patterns can be seen in figure 3. EpCAM was expressed in 8 of 11 HFE cases and in only 2 of 11 non-HFE controls (p=0.030). SALL4 was expressed in 6 of 11 HFE cases and in 2 of 11 non-HFE controls (p=0.183). EpCAM/SALL4 coexpression was only found in HFE-HCC (5/11 vs 0/11, p=0.035). A broad panel of EMT markers including CD44, vimentin, e-cadherin, CK18 and β-catenin were evaluated. No significant differences in these markers were found between the cohorts.

**Table 3:** Marker expression in HFE and non-HFE groups

	Process	Non-HFE, n /N (%)	HFE, n/N (%)	P value
EpCAM	CSC	2/11 (18)	8/11 (72)	0.030
SALL4	CSC	2/11 (18)	6/11 (55)	0.183
EpCAM/SALL4 coexpression	CSC	0/11 (0)	5/11 (45)	0.035
CD44	CSC and EMT	7/11 (64)	9/11 (81)	0.635
Vimentin	EMT	1/11 (9)	6/11 (55)	0.063
β-catenin	EMT	10/11 (91)	6/11 (55)	0.149
E-cadherin*	EMT	8/11 (72)	8/11 (72)	1.000
CK18*	EMT	3/11 (27)	8/11 (73)	0.086

* eExpression ≥5 on a scale of 0–9.

CSCcancer stem cellsEMTepithelial–mesenchymal transitionEpCAMepithelial cell adhesion moleculeHFEhaemochromatosisSALL4spalt-like transcription factor 4

## Discussion

Patients, particularly men, with HFE are at an increased risk of developing HCC compared with those without HFE.^2^ Some studies suggest that HCC which develops in patients with HFE behaves in a more aggressive fashion compared with those without HFE-HCC. Indeed, in the context of OLT, combined data from the UK, Australia and New Zealand suggest that patients with HFE have inferior 1-year and 5-year survival rates of 72% and 55%, respectively, compared with non-HFE graft recipients.^3^ In this study, HCC recurrence (5 of 11) represented a significant proportion of the deaths in the HFE arm. Notably, however, four of these patients underwent transplantation despite the presence of large tumours (7 cm, 9 cm, 10 cm and 12 cm) as there were no consensus criteria available at the time. Data analysis from 12 US centres appears to support these concerns, with poor 5-year survival in HFE OLT recipients of 34%.^4^ In contrast, data from King’s College London and the United Network for Organ Sharing suggest that HFE and non-HFE OLT recipients have comparable survival, with HCC recurrence contributing to a minority of patient deaths.^21 22^ These studies are limited by a large number of confounding variables, which might explain the variation in their findings, including baseline tumour number, tumour type and volume at the time of listing, AFP level, type of locoregional therapy used as a bridge to transplantation, and length of time on the waiting list. In our study we focused only on patients offered curative therapies, including thermal ablation and resection. We excluded patients listed for liver transplantation because of the potential bias that would be introduced, as outlined previously. We selected a comparator group that comprised patients who developed HCC on a background of ALD or NASH, whose outcomes following non-transplant curative therapy have been shown to be similar.^23^ We show that HFE-HCC is more aggressive as evidenced by a shorter time to progression and progression to more advanced disease during our short study follow-up.

In order to explain the difference in clinical outcomes, we sought to examine the expression of EMT and CSC markers which have previously been shown to promote tumour progression.^6^,^12^ EpCAM and SALL4 are established CSC markers absent in normal mature hepatocytes. Their expression in HCC has been associated with more aggressive tumour behaviour.^6 8^ Our results have demonstrated that patients with HFE-HCC have a significantly higher incidence of these markers when compared with those without HFE. A notable finding in this study was the presence of combined tumour subtypes (HCC-CCA) found in patients with HFE. These are rare tumours. A large study looking at their prevalence in patients diagnosed with HCC and CCA demonstrated a frequency of <1%.^24^ Our local review of combined HCC-CCA over a 20-year period identified only seven cases, of which three are included in this study. We therefore observe a far higher than expected proportion of HCC-CCA tumours in the HFE-HCC group (3 of 11) than might be expected. In contrast, there were no combined tumour subtypes found within the non-HFE-HCC samples. The origins of HCC-CCA tumours are not fully understood but are thought to arise from transformed multipotent liver progenitor cells,^25^ and are thus enriched with CSC markers.^26^ In addition to this, we also describe an almost unique case of combined HCC-yolk sac liver tumour.^27^ Extragonadal yolk sac tumours are thought to be derived from tumour-initiating stem cells.^28^ Indeed, a large study describing the immunohistochemical features of extragonadal yolk sac tumours identifies SALL4 as a sensitive marker.^29^ These observations support our hypothesis that iron accumulation in patients with HFE applies selective pressure on the liver stem cell niche, driving stem cell tumour development (with CSC expression) and combined tumour formation. We did not examine the mechanism of iron-related tumourigenesis. However, there are published data suggesting a direct impact on p53 activity,^30^ as well as increased oxidative stress and mitochondrial dysfunction leading to HCC development.^31^

There are a number of limitations in our study. We excluded all patients who were listed for liver transplantation in the clinical arm. The reason for this was to avoid innumerable variables which would limit the ability to draw robust conclusions in this study. As such, we included 12 patients who underwent curative therapies, including resection or thermal ablation, in the HFE group. We selected a comparator group that underwent similar therapy on a background of either ALD or NASH. There were no differences between the cohorts for variables which are normally associated with tumour progression and prognosis, including age, sex, BCLC stage of disease, presence of cirrhosis and AFP level. We did not have genetic confirmation of HFE in all patients in the HFE cohort. This could potentially have led to the inclusion of individuals without genetic HFE in the cohort of interest, and in turn may have tempered our results. The study follow-up time was also short; the median follow-up was approximately 2 years in both groups. Although we were unable to demonstrate a difference in survival between the groups, we feel it is likely that such a difference would have emerged over time. This will be the focus of further study. We were unable to use tissue from the clinical cohort for the immunohistochemical study as patients undergoing thermal ablation did not have protocol biopsy prior to therapy. For the immunohistochemical assessment, patients who underwent resection or OLT for HFE-HCC were identified with sufficient available tissue. These were carefully matched to a cohort of patients with variables normally associated with disease progression prior to therapy: age, sex, BCLC stage, lymphovascular invasion, cirrhosis, and tumour number and diameter. Given the strict matching criteria, we were unable to select a comparator group consistent only of either ALD or NASH aetiologies. There was not enough material available to perform genetic studies, which would have helped validate our outcomes.^32^ Additionally, we would have liked to assess combined expression levels of HN1, RAN, RAMP3, KRT19 and TAF9 in patients with HFE-HCC and those without HFE,^33^ and this is now the focus of further study in our department.

In summary, this study demonstrates that patients with HFE who develop HCC suffer negative clinical outcomes and have more aggressive tumour biology. One possible impact of this study is that patients with HFE with curative HCC might be considered for OLT at an earlier stage, rather than resection or thermal ablation. The increased expression of CSCs and the prevalence of combined tumour subtypes in the HFE cohort suggest an independent effect of iron on progenitor cell proliferation and differentiation within the liver. The findings outlined in this study highlight the need for early diagnosis and treatment of HFE-HCC. Moreover, the role of iron in liver tumour development through the activation of CSC pathways requires further study.

## supplementary material

10.1136/jcp-2022-208679 online supplemental file 1

## Data Availability

Data are available upon reasonable request.
