# Supplementary material for: Tumour stemness and poor clinical outcomes in haemochromatosis patients with hepatocellular carcinoma
Source: J Clin Pathol. 2023 May 30;77(10):e208679. doi: 10.1136/jcp-2022-208679 (PMC11503110; doi:10.1136/jcp-2022-208679)
Supplement: online supplemental file 1 [file jcp-77-10-s001.pdf]

Tumour stemness and poor clinical outcomes in haemochromatosis patients with hepatocellular carcinoma

Daniel M Di Capua[1]* William Shanahan[2]*

Michele Bourke[2]

Navneet Ramlaul[2] Josh Appel[2]

Aoife Canney[3]

Neil G Docherty[4,5]

Erinn McGrath[1]

Eabha Ring[2]

Fiona Jones[2]

Marie Boyle[2]

Janet McCormack[6]

Tom Gallagher[8]

Emir Hoti[8]

Niamh Nolan[1]

John D Ryan[9]

Diarmaid D Houlihan[1]^

Aurelie Fabre1,4,6^

1-HIstopathology, St Vincent's University Hospital, Dublin, Ireland

2-Liver Unit, St Vincent's University Hospital, Dublin, Ireland

3-Histopathology, University Hospital Galway, Galway, Ireland

4-University College Dublin School of Medicine, Dublin, Ireland

5- Diabetes Complications Research Centre, Conway Institute, University College Dublin, Dublin, Ireland

6- Research Pathology Core, Conway Institute, University College Dublin, Dublin, Irelandx

7- Hepatobiliary Service, St Vincent's University Hospital, Dublin, Ireland

8- Hepatology Unit, Beaumont Hospital, Dublin

9- RCSI University of Medicine and Health Sciences, Dublin

[*, ^ authors contributed equally to this work]

SUPLLEMENTARY DATA

Supplementary Methods:
Immunohistochemistry Antibody specification

Supplemental Table 1: Antibody characteristics, product information and primary incubation information

	PROVIDER/ PRODUCT CODE	ANTIGEN RETRIEVAL	POSITIVE CONTROL	DILUTION FACTOR	INCUBATION TIME
**B-CATENIN**	Dako/IR702	Target Retrieval Solution High pH	Liver	RTU	20 min
**CK18**	Dako/IR618	Target Retrieval Solution High pH	Liver	RTU	20 min
**E-CADHERIN**	Dako/IR059	Target Retrieval Solution High pH	Liver	RTU	20 min
**VIMENTIN**	Dako/IR630	Target Retrieval Solution High pH	Liver	RTU	20 min
**EPCAM**	Dako/IR637	Target Retrieval Solution Low pH	Colon	RTU	20 min

**CD44**	Dako/M7082	Target Retrieval Solution Low pH	Tonsil	1:50	30 min
**SALL4**	Abcam/ab57577	Target Retrieval Solution Low pH	Testis	1:250	30 min

This table highlights product information, including provider and product code, for each antibody used in this study. Immunohistochemical staining parameters of heat mediated antigen retrieval solution for the PT link bath, dilution factor, and incubation can be found in this table as well. Each antibody assay performed on the Dako Autostainer 48 included the antibody appropriate positive control highlighted above.

Evaluation of immunohistochemical staining

The American Society of Clinical Oncology/College of American Pathologists algorithm for evaluation of HER2 immunohistochemical protein expression by tumour cells in breast cancer[1] was employed for the assessment of EpCAM staining by HCC tumour cells (Table 2).

Supplemental Table 2: Immunohistochemical scoring system for evaluation of EpCAM expression.

SCORE	IMMUNOHISTOCHEMICAL STAINING
**0**	No staining observed<br>*Or*<br>Faint incomplete membranous staining involving ≤10% of tumour cells
**1+**	Faint incomplete membranous staining involving >10% of tumour cells
**2+**	Incomplete and/or circumferential weak/moderate membranous staining involving >10% of tumour cells<br>*Or*<br>Intense complete circumferential staining involving ≤ 10% of tumour cells
**3+**	Intense, complete circumferential membranous staining involving >10% of tumour cells

Adapted for membranous EpCAM staining by HCC tumour from the he American Society of Clinical Oncology/College of American Pathologists algorithm evaluation of HER2 immunohistochemical protein expression by tumour cells in breast cancer[1].

Scoring systems were devised for semi-quantitative assessment of immunohistochemical staining for the nominated antibody panel. The algorithms were based on previously validated methods successfully applied in other studies of liver cancer[2] or widely used in clinical practice[1] (Supplemental Figure 1). A scoring algorithm incorporating distribution and intensity of immunoreactivity was applied to CD44, SALL4, e-cadherin and CK18 immunoassays. Binary scoring was applied to vimentin where any staining was considered positive and β-catenin where nuclear staining was considered positive.

Demonstration of representative panels for the array of scores that can be obtained through the distribution and intensity scoring algorithm. Images used to compile this figure consist of HCC sections stained with CD44. A distribution (D-score) and intensity (I-score) value was determined

for CD44, SALL4, e-cadherin, and CK18 immunohistochemical assays as outlined above. Distribution of staining refers to the percentage of immunoreactive tumour cells per tissue section. Intensity of immune-positive tumour cells was graded from mild-marked. The product of the D-score and the I-score provided a final immunoreactivity score per assay.

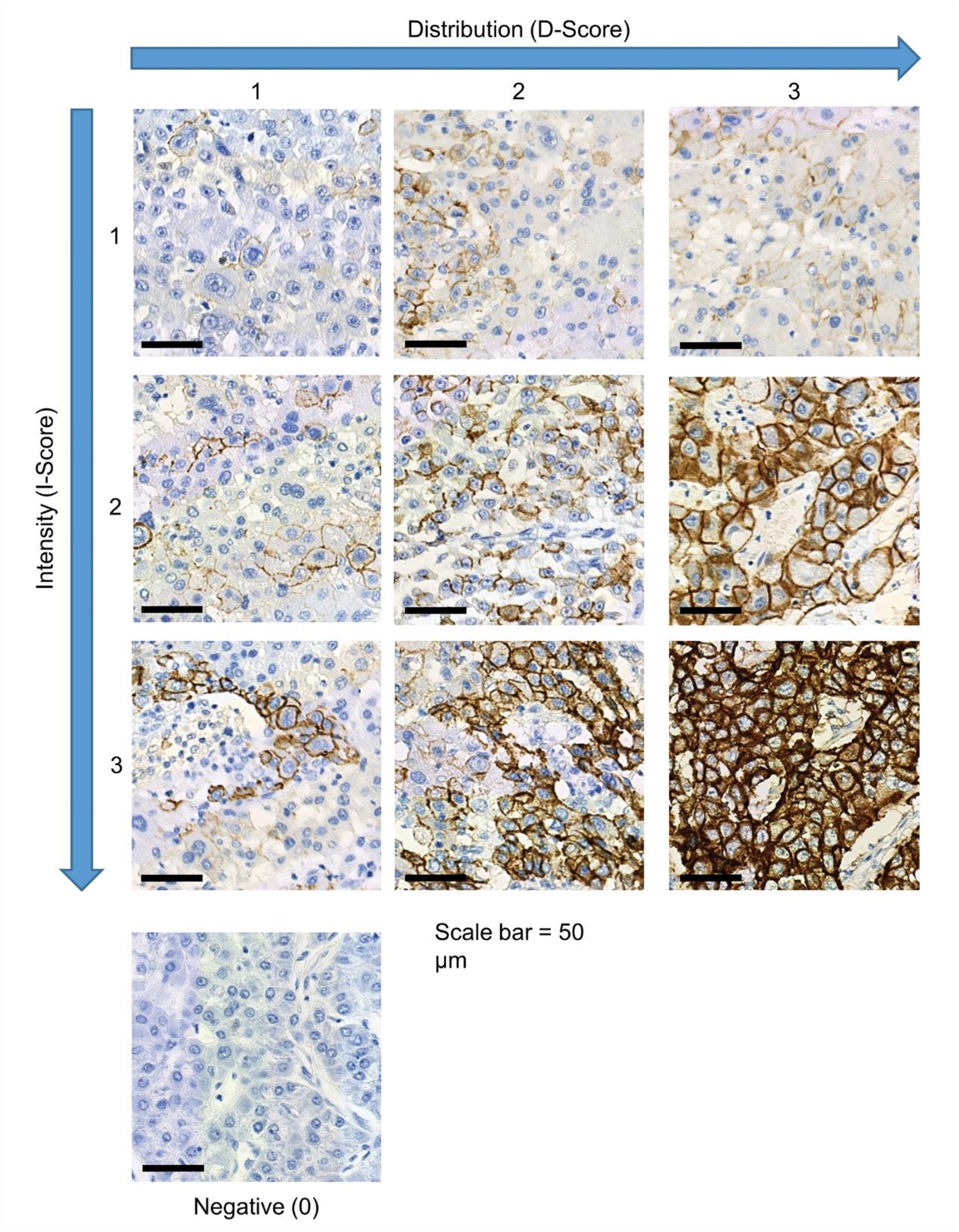

Supplemental Figure 1: Distribution and intensity immunohistochemical scoring algorithm for CD44, SALL4, e-cadherin and CK18.

Supplementary References:

1. Wolff AC, Hammond MEH, Hicks DG, et al. Recommendations for human epidermal growth factor receptor 2 testing in breast cancer: American society of clinical oncology/college of American pathologists clinical practice guideline update. *Arch Pathol Lab Med* 2014;138:241–256.
2. Endo K, Terada T. Protein expression of CD44 (standard and variant isoforms) in hepatocellular carcinoma: Relationships with tumor grade, clinicopathologic parameters, p53 expression, and patient survival. *J Hepatol* 2000;32:78–84.
